# Association between *IL-1B (-511)/IL-1RN* (VNTR) polymorphisms and type 2 diabetes: a systematic review and meta-analysis

**DOI:** 10.7717/peerj.12384

**Published:** 2021-10-25

**Authors:** Juan Jiao, Zhaoping Wang, Yanfei Guo, Jie Liu, Xiuqing Huang, Xiaolin Ni, Danni Gao, Liang Sun, Xiaoquan Zhu, Qi Zhou, Ze Yang, Huiping Yuan

**Affiliations:** 1 The Key Laboratory of Geriatrics, Beijing Institute of Geriatrics, Institute of Geriatric Medicine, Chinese Academy of Medical Science, Beijing Hospital/National Center of Gerontology of National Health Commission, P.R. China; 2 Department of Clinical Laboratory, the Seventh Medical Center, Chinese PLA General Hospital, Beijing, China; 3 Department of Respiratory and Critical Care Medicine, Beijing Hospital, National Center of Gerontology; Institute of Geriatric Medicine, Chinese Academy of Medical Sciences, P.R. China; 4 Peking University Fifth School of Clinical Medicine (Beijing Hospital), Beijing, China

**Keywords:** IL-1B (-511), IL-1RN (VNTR), Polymorphism, Type 2 diabetes mellitus, Meta-analysis

## Abstract

Interleukin-1 (IL-1) plays an essential role in the immune pro-inflammatory process, which is regarded as one of many factors in the development of type 2 diabetes mellitus (T2DM). Several case-control studies have illustrated the association of the *IL-1B* (-511) (rs16944, Chr 2:112,837,290, C/T Intragenic, Transition Substitution) and *IL-1RN* (VNTR) (gene for IL-1 receptor antagonist, IL-1RA, 86 bp tandem repeats in intron 2) polymorphisms with T2DM risk. However, the results were inconsistent and inconclusive. We performed a meta-analysis (registry number: CRD42021268494) to assess the association of the *IL-1B (-511)* and *IL-1RN* (VNTR) polymorphisms with T2DM risk. Random-effects models were applied to calculate the pooled ORs (odds ratios) and 95% CIs (confidence intervals) to test the strength of the association in the overall group and subgroups stratified by ethnicity, respectively. Between-study heterogeneity and publication bias were evaluated by the *Q*-test, *I^2^* statistic, Harbord test, and Peters test accordingly. Sensitivity analyses were also performed. A total of 12 publications evaluating the association of *IL-1B (-511)* and *IL-1RN* (VNTR) polymorphisms with the risk of T2DM development were included. The meta-analysis showed that *IL-1RN* (VNTR) was related to the increasing development of T2DM risk in the recessive model (OR = 1.62, 95% CI [1.09–2.42], *P_het_* = 0.377, *P_z_* = 0.018) and in the homozygous model (OR = 2.02, 95% CI [1.07–3.83], *P_het_* = 0.085, *P_z_* = 0.031), and the IL-1RN 2* allele was found a significant association with evaluated T2DM risk in all ethnicities (OR = 2.08, 95% CI [1.43–3.02], *P_het_* < 0.001, *P_z_* < 0.001) and in EA (OR = 2.01, 95% CI [1.53–2.66], *P_het_* = 0.541, *P_z_* < 0.001). Moreover, stratification by ethnicity revealed that *IL-1B (-511)* was associated with a decreased risk of T2DM in the dominant model (OR=0.76, 95% CI [0.59–0.97], *P_het_* = 0.218, *P*_z_ = 0.027) and codominant model (OR = 0.73, 95% CI [0.54–0.99], *P_het_* = 0.141, *P_z_* = 0.040) in the East Asian (EA) subgroup. Our results suggest that the IL-1RN 2* allele and 2*2* homozygous polymorphism are strongly associated with increasing T2DM risk and that the *IL-1B (-511) T* allele polymorphism is associated with decreasing T2DM risk in the EA subgroup.

## Introduction

The latest data from the 9th edition of the IDF Diabetes Atlas show that approximately 463 million adults (20–79 years) have diabetes, and by 2045, the number will rise to 700 million. Type 2 diabetes mellitus (T2DM) is the most prevalent type of diabetes (>90% of diabetes). The proportion of T2DM patients is increasing in most countries, especially in low- and middle-income countries such as China and India (https://www.idf.org/aboutdiabetes/what-is-diabetes/facts-figures.html). Growing evidence suggests that the risk of T2DM is associated with various factors, such as genetics, ethnicity, environment, and lifestyle. In particular, genetic susceptibility seems to play an essential role in the pathogenesis of T2DM (Hivert, Vassy & Meigs, 2014).

T2DM occurs when β-cell function progressively deteriorates and fails to compensate for insulin resistance, partly due to the demise of pancreatic β-cells through apoptosis (Donath & Halban, 2004). The chronic and low-grade inflammation in metabolic organs including the liver, brain, pancreas and adipose tissue is known as metaflammation (metabolism-induced inflammation), which can be widely observed in T2DM (Hotamisligil, 2006; Hotamisligil, 2017). Increasing evidence has shown that metaflammation plays a vital role in the development of T2DM and its cardiovascular complications (Hotamisligil, 2017) and pro-inflammatory cytokines, especially interleukin-1β (IL-1β), play a key role in this process (Dinarello, Donath & Mandrup-Poulsen, 2010; Fève & Bastard, 2009; Sathyapalan & Atkin, 2011). Moreover, the increased level of IL-1β in human pancreatic cells due to the elevated glucose concentrations and decreased level of IL-1 receptor antagonist (IL-1Ra) in islets of T2DM patients result in impaired insulin secretion, decreased cell proliferation, and apoptosis of β-cells (Böni-Schnetzler & Donath, 2013). A previous observational study demonstrated that a combined elevation of IL-1β and IL-6 was associated with a roughly threefold increased risk of T2DM, and IL-1β might induce insulin resistance *via* activating the IκB kinaseβ (Spranger et al., 2003). Furthermore, CANTOS and TRACK trials have evaluated the role of IL-1 inhibition in the treatment of several inflammatory disorders, such as coronary artery disease, T2DM and rheumatoid arthritis (Qamar & Rader, 2012; Ruscitti et al., 2019). According to the result from CANTOS trials, canakinumab (a fully humanized monoclonal antibody) could selectively block IL-1β, and its efficacy in suppressing both levels of inflammatory markers and clinical symptoms in patients with autoinflammatory disease such as T2DM and impaired glucose tolerance has been observed in this trial (Aday & Ridker, 2018; Rissanen et al., 2012). Therefore, the genetic polymorphisms that regulate the expression levels of IL-1β and IL-1Ra might have an essential impact on the interindividual differences in T2DM.

Consisting of three linked genes and mapping to chromosome 2q13-24, IL-1 cytokine genes encode IL-1α, IL-1β and IL-1Ra. Both IL-1α and IL-1β are pro-inflammatory cytokines, while IL-1Ra can inhibit inflammation by competing for receptor binding (Dinarello, 2000). In the promoter region, *IL-1B (-511)* (rs16944, Chr 2:112,837,290, C/T Intragenic, Transition Substitution) has a bi-allelic polymorphism at position -511, representing the C/T transition (Kristiansen et al., 2000). *IL-1RN* (VNTR) (gene for IL-1 receptor antagonist, IL-1RA, 86 bp tandem repeats in intron 2) has five different alleles: 1* (four repeats), 2* (two repeats), 3* (five repeats), 4* (three repeats) and 5* (six repeats) (Tarlow et al., 1993). IL-1RN 1* (four repeats) and IL-1RN 2* (two repeats) are the most common, whereas the others occur much less frequently (<5%) (Santtila, Savinainen & Hurme, 1998). Many genetic association studies have been performed to estimate the relationship of the *IL-1B (-511)* and *IL-1RN* (VNTR) polymorphisms with T2DM risk (Achyut et al., 2007; Muktabhant et al., 2013). Several results suggest that there is no relationship between them (Borilova Linhartova et al., 2019; Muktabhant et al., 2013), whereas Achyut et al., (2007) showed that both the *IL-1B (-511)* and *IL-1RN* (VNTR) polymorphisms were associated with susceptibility to T2DM as well as complications, and he indicated that *T2* (*IL-1*β −511T/IL-1RN*2) haplotype was associated with a roughly twofold increased risk of T2DM. Considering the small sample sizes (*n* <  600) and varying population characteristics (ethnic differences) in different studies, the results are conflicting. To illustrate the potential association of the *IL-1B (-511)* and *IL-1RN* (VNTR) polymorphisms with T2DM risk, we conducted a meta-analysis including 12 reported publications.

## Materials and Methods

This systematic review was conducted following the Preferred Reporting Items for Systematic Reviews and Meta-Analysis (PRISMA) statement guidelines (Moher et al., 2009). A protocol was registered before commencing this review on PROSPERO (CRD42021268494).

### The search strategy

The online databases we searched included PubMed, Web of Science, CNKI (China National Knowledge Infrastructure) and Wanfang. We identified relevant articles reporting the association of *IL-1RN* (VNTR) and *IL-1B (-511)* polymorphisms with the risk of T2DM in the medical literature through the end of September 12, 2020. This study used a combination of the following search terms: “Type 2 Diabetes Mellitus” or “T2DM” or “interleukin-1” or “IL-1” or “polymorphism” or “genetics” or “association”. In addition, we also conducted a hand search to identify relevant data in references from retrieved articles. Juan Jiao and Zhaoping Wang performed the Search Strategy. In case of disagreement, it will be settled by a third assessor’s evaluation and discussed until a consistent result was reached.

### The inclusion and exclusion criteria

All studies included in this analysis met the following criteria: (1) the study revealed the relationship of *IL-1B (-511)* and *IL-1RN* (VNTR) with T2DM risk; (2) the design was a “case-control study”; (3) the research subjects were humans with T2DM; (4) the publication language was English or Chinese; and (5) the study provided sufficient data to estimate ORs (odds ratios) and 95% CIs (confidence intervals). The major exclusion criteria were as follows: (1) duplicate data; (2) randomly chosen controls; (3) non-research articles; and (4) insufficient reporting of data.

### Data extraction

All data were systematically reviewed and extracted by two investigators (ZPW and DNG) according to a standardized form, and then all of the following information was collected in an electronic database: the first author’s name, year of publication, country of origin, ethnicity, total number of cases/controls, genotyping method, diagnostic criteria of T2DM, genotypic frequencies and language of the report. Disagreement was settled by a third assessor’s evaluation and discussed until a consistent conclusion was agreed.

### Assessment of quality score

We assessed the quality score of identified publications based on the Newcastle Ottawa Scale (NOS) (http://www.ohri.ca/programs/clinical_epidemiology/oxford.asp). And the NOS involved “selection”, “comparability”, and “estimation of outcomes or exposures”. The scores ranged from 0 to 9, and the score of “high quality study” was ≥ 6 (detailed scores see Table S1) (Lo, Mertz & Loeb, 2014). And the STREGA (Strengthening the REporting of Genetic Association) system was also performed to assessed the methodological quality of all included studies, which includes 22 items with scoring from 0 to 22 (detailed scores see Table S2) (Little et al., 2009). And the score of high quality was 18–22; the score of moderate-high quality was 13–17; the score of low quality was 0–12 (Duan et al., 2018).

**Table 1:** Characteristics of Identified Studies on *IL-1B (-511)*/*IL-1RN(VNTR)* polymorphism and the risks of T2DM.

Author	Year	Country/Ethnicity	Diagnostic criteria	Genotyping method	Number		Language	IL-1 gene	*QA*	*HWE*
					Cases	Controls				
Zhang Jian	2004	China/EA	NA	PCR-RFLP	106	198	Chinese	*IL-1RN*	7	1.00
Zhang Ping-an	2004	China/EA	WHO, 1999	PCR-RFLP	106	247	Chinese	*IL-1RN*	8	0.70
Zhou Jian-zhong	2010	China/EA	WHO, 1999	PCR-RFLP	72	97	Chinese	*IL-1RN*	7	0.23
Petra Borilova Linhartova	2019	Czech Republic/Caucasian	NA	PCR-RFLP	380	212	English	*IL-1RN*	8	1.00
Alexandra I. F. Blakemore	1995	North of England/Caucasian	WHO, NA	PCR	117	248	English	*IL-1RN*	7	0.61
B.R Achyut	2006	North Indian/SA	WHO, 1999	PCR-RFLP	200	223	English	*IL-1RNIL-1B (-511)*	8	0.100.16
Liu Chang	2014	China/EA	WHO, 1999	PCR-RFLP	583	366	Chinese	*IL-1B (-511)*	7	1.00
Cao Yong	2013	China/EA	WHO, 1999	PCR-RFLP	268	263	Chinese	*IL-1B (-511)*	8	0.06
Lin Neng-bo	2016	China/EA	WHO, 1999	PCR-RFLP	286	327	Chinese	*IL-1B (-511)*	8	0.08
Natalie E. Doody	2017	North Indian/SA	WHO, 1999	PCR-RFLP	202	203	English	*IL-1B (-511)*	8	0.87
Benja Muktabhant	2013	Thailand/SEA	NA	PCR-RFLP	90	30	English	*IL-1B (-511)*	7	0.16
Safaa I. Tayel	2018	Egypt/NA_1_	ADA, NA	TaqMan	50	30	English	*IL-1B (-511)*	8	1.00

**Notes.**

T2DM Type 2 diabetes mellitus EA East Asian SA South Asian SEA South East Asian NA Not Available (the diagnosis of T2DM was originally based on the presence of clinical symptoms and biochemical) NA1 North African ADA American Diabetes Association (fasting blood glucose ≥126 mg/dL or 2 h blood glucose after overload with 75 g of glucose ≥ 200 mg/dL in oral glucose tolerance test (OGTT) or glycated hemoglobin (HbA1c) ≥6.5 in patients with classic symptoms of hyperglycemia) WHO World Health Organization (fasting glucose levels [7.0 mmol/L or 126 mg/dL] after a minimum 12-h fast or 2-h post glucose level (oral glucose tolerance test or 2-h OGTT) [11.1 mmol/L or 200 mg/dL] on more than one occasion) QA Quality Assessment HWE Hardy-Weinberg Equilibrium

### Data analysis

We conducted this analysis and entered data with Stata software, version 15.0 (Stata Corp., College Station, TX, USA). We used original genotypic distribution data without any adjustment to measure the strength of the association between *IL-1B (-511)*/*IL-1RN* and T2DM risk by ORs (odds ratios) with 95% CIs (confidence intervals) under dominant, recessive, additive (overdominant+codominant) and homozygous models. The distributions of genotype frequencies of controls were all consistent with Hardy-Weinberg Equilibrium (*HWE*) (*P* > 0.05, Table 1). Between-study heterogeneity among the studies was tested with Cochran’s *Q* and the *I*^2^ test statistics. *I*^2^ was calculated based on the formula *I*^2^ = 100% ×(*Q*−*df*)/*Q*. Heterogeneity was regarded as significant when *P*_*het*_ < 0.1 or *I*^2^ > 50%. For those with significant heterogeneity, we performed stratified analyses to examine the statistical significance of the difference in ORs according to ethnicity (East Asian, South Asian, North African and Caucasian). We used the random-effects model (DerSimonian and Laird method) which was combined by applying inverse variance-weighted meta-analysis to calculate the pooled OR and 95% CI, with *P*_*z*_ < 0.05 considered statistically significant (DerSimonian & Laird, 1986). When the heterogeneity was significant, sensitivity analyses were conducted to evaluate the influence of each single study by omitting one study at a time and checking the pool effect size for the remainder of the studies. Finally, the Harbord test and Peters test were used when fewer than ten articles were included, as they are more sensitive for assessing publication bias, with *P* < 0.1 assuming that the bias was statistically significant.

## Results

### Included studies

**Figure 1:**
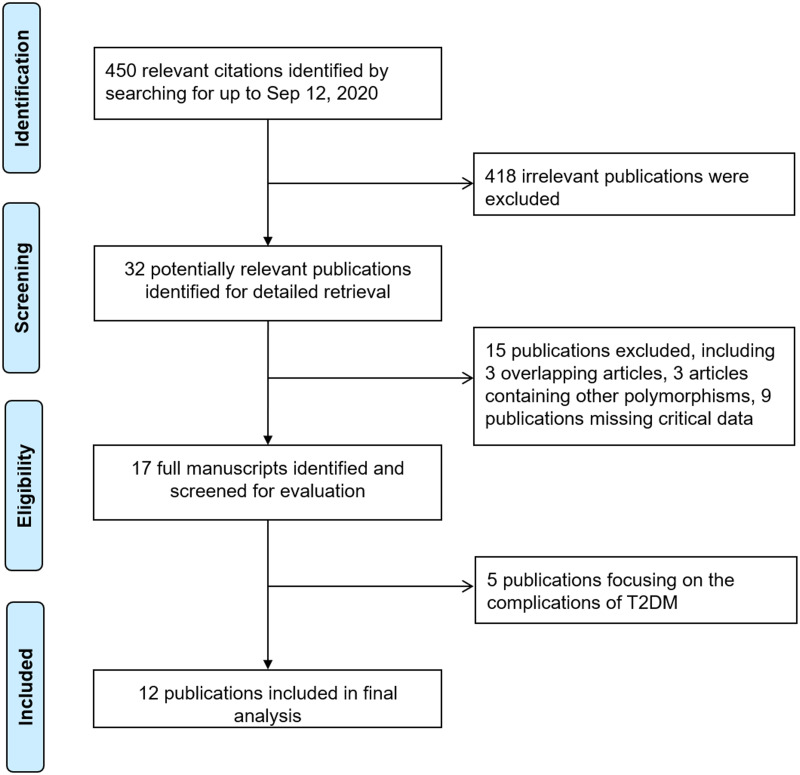
Flowchart of study identification process.

Figure 1 shows the detailed process of selecting and assessing eligible studies. We identified 450 publications *via* the initial keyword search. After screening, 12 publications met our inclusion criteria (Achyut et al., 2007; Blakemore et al., 1996; Borilova Linhartova et al., 2019; Cao et al., 2013; Doody et al., 2017; Lin et al., 2016; Liu et al., 2014; Muktabhant et al., 2013; Tayel et al., 2018; Zhang, Xiao & Li, 2004a; Zhang et al., 2004b; Zhou et al., 2010), and 438 studies were excluded for containing duplicate data, missing critical data, and being review papers or studies that were not related to T2DM. Table 1 illustrates the characteristics of all included publications in this meta-analysis. In terms of polymorphisms and diseases, six identified case-control studies, comprising 981 cases and 1,225 controls, regarded *IL-1RN* (VNTR) to evaluate its association with T2DM risk (Achyut et al., 2007; Blakemore et al., 1996; Borilova Linhartova et al., 2019; Zhang, Xiao & Li, 2004a; Zhang et al., 2004b; Zhou et al., 2010), and seven identified case-control studies, including 1,679 cases and 1,442 controls, tested the association between *IL-IB (-511)* and T2DM risk (Achyut et al., 2007; Cao et al., 2013; Doody et al., 2017; Lin et al., 2016; Liu et al., 2014; Muktabhant et al., 2013; Tayel et al., 2018). Stratified by ethnicity, six publications were performed in East Asian (EA) populations (Cao et al., 2013; Lin et al., 2016; Liu et al., 2014; Zhang, Xiao & Li, 2004a; Zhang et al., 2004b; Zhou et al., 2010), two studies were undertaken in South Asian (SA) populations (Achyut et al., 2007; Doody et al., 2017), two studies were conducted in Caucasian populations (Blakemore et al., 1996; Borilova Linhartova et al., 2019), and the remaining studies were completed in Southeast Asian (SA) (Muktabhant et al., 2013) and North African (NA_1_) populations (Tayel et al., 2018).

### Individual polymorphism meta-analysis

The genotypic distributions of the *IL-1B (-511)* and *IL-1RN* (VNTR) polymorphisms are shown in Table 2. Table 3 shows the meta-analysis results for these two polymorphisms.

**Table 2:** The IL-1 polymorphism distribution in cases and controls. Table 2 shows the genotypic distributions of the *IL-1B (-511)* and *IL-1RN (VNTR)* polymorphisms.

Gene	Case					Control				
***IL-1B (-511)***	***C***	***T***	***CC***	***TC***	***TT***	***C***	***T***	***CC***	***TC***	***TT***
Cao Yong	302	270	78	129	61	332	322	52	147	64
Liu Chang	634	532	171	292	120	385	347	101	183	82
B.R Achyut	108	292	9	90	101	175	271	29	117	77
Lin Neng-bo	302	270	85	132	69	332	322	76	180	71
Safaa I. Tayel	55	45	16	23	11	21	39	4	13	13
Natalie E. Doody	186	218	40	106	56	133	273	21	91	91
Benja Muktabhant	90	90	23	44	23	33	27	11	11	8
***IL-1RN***	1*	2*	1*/1*	1*/2*	2*/2*	1*	2*	1*/1*	1*/2*	2*/2*
B.R Achyut	227	234	60	107	20	340	92	138	64	14
Zhang Jian	192	34	88	16	2	360	36	163	34	1
Zhang Ping-an	192	34	88	16	2	450	44	204	42	1
Zhou Jian-zhong	107	71	36	35	1	153	41	58	37	2
Alexandra I. F. Blakemore	154	106	55	44	18	372	124	141	90	17
Petra Borilova Linhartova	569	289	220	129	31	303	121	108	87	17

**Table 3:** Meta-analysis of the relationship of the IL-1 polymorphisms with the risks of T2DM.

Gene	No. studies	Allele comparison				Genetic model comparison			
		OR (95% CI)	*P*_*het*_	*P*_*z*_		OR (95% CI)		*P*_*het*_	*P*_*z*_
*IL-1B (-511)*									
Total	7	0.89 (0.68–1.17)	<0.001	0.406		Dominant	0.84 (0.57–1.25)	0.001	0.395
						Recessive	0.89 (0.61–1.31)	<0.001	0.561
						Overdominant	0.92 (0.74–1.14)	0.065	0.422
						Codominant	0.85 (0.6–1.21)	0.011	0.375
						Codominant^#^	0.96 (0.69–1.34)	0.003	0.803
						Homozygote	0.82 (0.48–1.39)	<0.001	0.451
EA	3	0.89 (0.79–1.01)	0.606	0.072		**Dominant**	**0.76 (0.59–0.97)**	**0.218**	**0.027**
						Recessive	0.97 (0.79–1.20)	0.589	0.789
						Overdominant	0.82 (0.64–1.04)	0.162	0.094
						**Codominant**	**0.73 (0.54–0.99)**	**0.141**	**0.040**
						Codominant^#^	1.07 (0.86–1.34)	0.385	0.531
						Homozygote	0.80 (0.62–1.03)	0.571	0.081
SA	2	1.00 (0.33–2.98)	<0.001	0.997		Dominant	1.20 (0.18–7.84)	<0.001	0.853
SEA	1	1.22 (0.68–2.20)	–	0.503		Dominant	1.69 (0.70–4.07)	–	0.245
NA	1	**0.44 (0.23–0.85)**	**–**	**0.015**		Dominant	0.33 (0.10–1.10)	–	0.070
*IL-1RN*									
Total	6	**2.08 (1.43–3.02)**	**<0.001**	**<0.001**		Dominant	1.35 (0.79–2.31)	<0.001	0.275
						**Recessive**	**1.62 (1.09–2.42)**	**0.377**	**0.018**
						Overdominant	1.20 (0.72–1.99)	<0.001	0.485
						Codominant	1.28 (0.73–2.24)	<0.001	0.398
						Codominant^#^	1.38 (0.89–2.13)	0.351	0.155
						**Homozygote**	**2.02 (1.07–3.83)**	**0.085**	**0.031**
EA	3	**2.02 (1.53–2.66)**	**0.541**	**<0.001**		Dominant	1.11 (0.78–1.59)	0.525	0.558
						Recessive	2.30 (0.57–9.25)	0.470	0.243
						Overdominant	1.05 (0.72–1.54)	0.337	0.790
						Codominant	1.06 (0.74–1.53)	0.371	0.749
						Codominant^#^	2.25 (0.53–9.55)	0.356	0.270
						Homozygote	2.42 (0.60–9.78)	0.554	0.216
SA	1	**3.81 (2.84–5.11)**	**–**	**<0.001**		Dominant	3.75 (2.48–5.67)	–	<0.001
Caucasian	2	1.60 (0.998–2.58)	0.02	0.051		Dominant	1.04 (0.54–2.02)	0.017	0.901

**Notes.**

CI confidence interval IL-1 interleukin-1 OR odds ratio *P*_*het*_ *P*-value for heterogeneity *P*_*z*_ *P*-value for overall effect SA South Asian SEA South East Asian EA East Asian NA North Africa

For *IL1B (-511)* polymorphism: dominant (*TT* + *TC* vs *CC*), recessive (*TT* vs *TC* + *CC*), overdominant (*CT vs CC+TT*), codominant (*CT vs CC*), codominant ^#^ (*CT vs TT*) and homozygote (*TT* vs *CC*).

For *IL1RN* (VNTR) polymorphism: dominant (2*2* + 2*1* vs 1*1*), recessive (2*2* vs 2*1* + 1*1*), overdominant (2*1* vs 1*1*+2*2*), codominant (2*1* vs 1*1*), codominant ^#^ (2*1*vs 2*2*) and homozygote (2*2* vs 1*1*).

**Figure 2:**
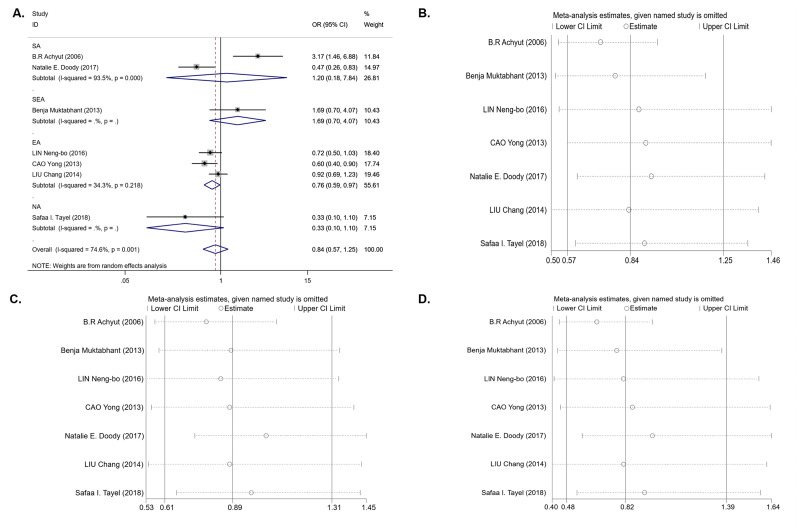
An overall forest plot of *IL-1B (-511)* associated with T2DM risk in the dominant model (*TT* + *TC vs CC*) and sensitivity analyses. (A) Forest plot of *IL-1B (-511)* associated with T2DM in the dominant model; (SA: South Asian, SEA: South East Asian, EA: East Asian, NA: North Africa). (B) Sensitivity analysis for *TT* + *TC vs CC* associated with T2DM in the overall meta-analysis. (C) Sensitivity analysis for *TT vs TC* + *CC* associated with T2DM in the overall meta-analysis. (D) Sensitivity analysis for *TT vs CC* associated with T2DM in the overall meta-analysis.

For the *IL-1B (-511)* polymorphism, seven studies with 1,679 cases and 1,442 controls were included in the meta-analysis. The results of the pooled analysis suggest that the *IL-1B (-511)* polymorphism is not significantly associated with T2DM risk in all study subjects under the dominant model (OR = 0.84, 95% CI [0.57–1.25], *P*_*het*_ = 0.001, *P*_*z*_ = 0.395) (Table 3 and Fig. 2A), recessive model (OR = 0.89, 95% CI [0.61–1.31], *P*_*het*_ < 0.001, *P*_*z*_ = 0.561) (Table 3), and homozygous model (OR = 0.82, 95% CI [0.48–1.39], *P*_*het*_ < 0.001, *P*_*z*_ = 0.451) (Table 3). In the stratification analyses by ethnicity, we found that there was a significant association between the *IL-1B (-511)* polymorphism and decreasing T2DM risk in the EA population under the dominant model (OR = 0.76, 95% CI [0.59–0.97], *P*_*het*_ = 0.218, *P*_*z*_ = 0.027) (Table 3 and Fig. 2A) and codominant model (OR = 0.73, 95% CI [0.54–0.99], *P*_*het*_ = 0.141, *P*_*z*_ = 0.040) (Table 3), but no association was observed under the recessive model (OR = 0.97, 95% CI [0.79–1.20], *P*_*het*_ = 0.589, *P*_*z*_ = 0.789) or homozygous model (OR = 0.80, 95% CI [0.62–1.03], *P*_*het*_ = 0.571, *P*_*z*_ = 0.081). In other ethnic groups, no association between the *IL-1B (-511)* polymorphism and T2DM risk was found. Between-study heterogeneity was found in the dominant, recessive and homozygous models (*P*_*het*_ = 0.001, *P*_*het*_< 0.001, *P*_*het*_< 0.001) (Table 3).

**Figure 3:**
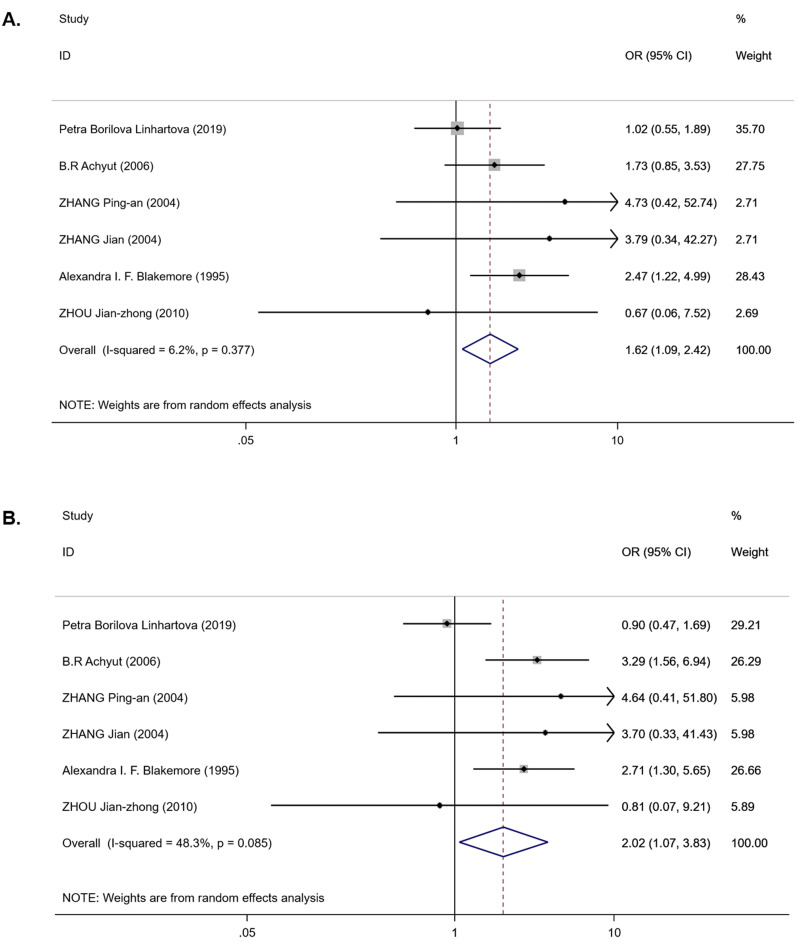
Forest plot of meta-analysis on *IL-1RN* (VNTR) and the risk of T2DM in the recessive model ( 2*2* vs 2*1* + 1*1*) and homozygous model (2*2* vs 1*1*). (A) Forest plot of *IL-1RN* (VNTR) associated with T2DM in the recessive model. (B) Forest plot of *IL-1RN* (VNTR) associated with T2DM in the homozygous model.

For the *IL-1RN* (VNTR) polymorphism, six publications were involved in the meta-analysis (980 cases and 1,225 controls). Overall, a significantly increased T2DM risk was found to be associated with 2* allele in all ethnicities (OR = 2.08, 95% CI [1.43–3.02], *P*_*het*_ < 0.001, *P*_*z*_ < 0.001) and in EA (OR = 2.01, 95% CI [1.53–2.66], *P*_*het*_ = 0.541, *P*_*z*_ < 0.001) (Table 3). Pooling data also revealed that this polymorphism is strongly associated with an increasing T2DM risk in the recessive model (OR = 1.62, 95% CI [1.09–2.42], *P*_*het*_ = 0.377, *P*_*z*_ = 0.018) (Table 3 and Fig. 3A) and homozygous model (OR = 2.02, 95% CI [1.07–3.83], *P*_*het*_ = 0.085, *P*_*z*_ = 0.031) (Table 3 and Fig. 3B), but there was no association in the dominant model (OR = 1.35, 95% CI [0.79–2.31], *P*_*het*_ < 0.001, *P*_*z*_ = 0.275) (Table 3). Between-study heterogeneity was found in the dominant model (*P*_*het*_ < 0.001) (Table 3).

### Sensitivity and publication bias analysis

We next performed sensitivity analysis on the association between IL-1β (-511) and T2DM. The results showed that Achyut’s study had an influence on the pooled OR in the IL-1β (-511)-dominant model (OR = 0.71, 95% CI [0.53–0.96]) and homozygous model (OR = 0.65, 95% CI [0.44–0.97]) (Figs. 2B, 2D) but did not affect the pooled OR in the recessive model (Fig. 2C). Achyut’s study was performed in a SA population; thus, there was no influence on the pooled OR in the EA population. Due to the lack of heterogeneity in IL-1RN recessive and homozygous models, sensitivity analysis was not performed.

**Figure 4:**
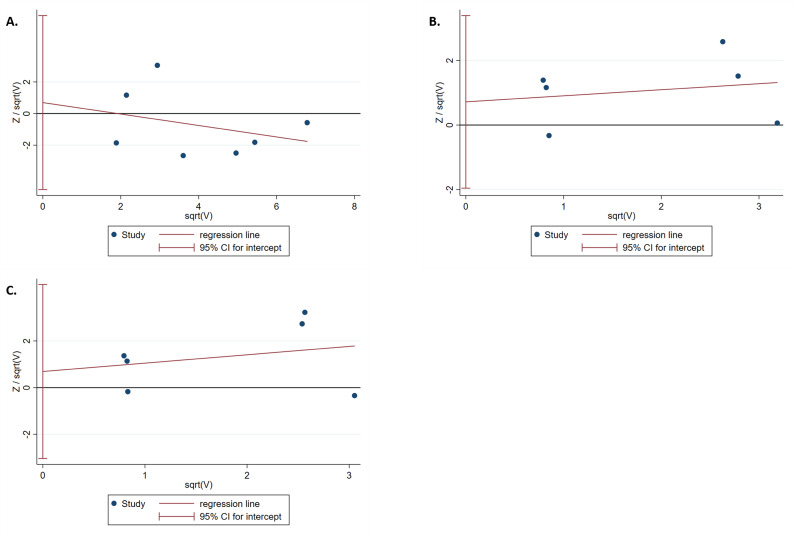
Harbord test for publication bias testing. (A) Publication bias of *IL-1B (-511)* associated with T2DM in the dominant model (*TT* + *TC vs CC*); (B) Publication bias of *IL-1RN* (VNTR) associated with T2DM in the recessive model (2*2* vs 2*1* + 1*1*); (C) Publication bias of *IL-1RN* (VNTR) associated with T2DM in the homozygous model(2*2* vs 1*1*).

The results of the Harbord test and Peters test showed no publication bias for *IL-1B (-511)* in the dominant model (*P*_*har*_ = 0.759 and *P*_*pet*_ = 0.881, respectively) (Fig. 4A). Moreover, no evidence of publication bias was found for *IL-1RN* (VNTR) in the recessive (*P*_*har*_ = 0.498 and *P*_*pet*_ = 0.495, respectively) or homozygous models (*P*_*har*_ = 0.634 and *P*_*pet*_ = 0.481, respectively) (Figs. 4B, 4C).

## Discussion

The *IL-1B (-511)* and *IL-1RN* (VNTR) polymorphisms have been reported to be related to the pathogenesis of T2DM (Ehses et al., 2009; Masters et al., 2010), and many case-control studies have illustrated the association of *IL-1B (-511)* and *IL-1RN* (VNTR) polymorphisms with T2DM risk. However, each individual study may not have been powerful, causing their results to be controversial. Given the above, we conducted the present meta-analysis to derive a more precise evaluation of the association of *IL-1B (-511)* and *IL-1RN* (VNTR) polymorphisms with T2DM risk. We found that (i) the *T* allele of *IL-1B (-511)* was related with a decreased T2DM risk in the EA subgroup; (ii) significantly elevated risk of developing T2DM was observed to be associated with the 2* allele in all ethnicities and EA subgroup, and its carriers of the *IL-1RN* (VNTR) polymorphism had an increased risk of developing T2DM among all ethnicities; (iii) compared to the 1*/1* homozygotes, the 2*/2* homozygotes of the *IL-1RN* (VNTR) polymorphism had an increased risk of T2DM development; and (iv) no significant association was observed between the *IL-1RN* (VNTR) polymorphism and T2DM risk in the EA subgroup. The results of sensitivity analysis showed that Achyut’s study affected the pooled ORs in the IL-1β (-511) dominant and homozygous models. However, Achyut’s study did not influence the pooled OR in the EA subgroup after stratification by ethnicity. Therefore, the result of the association between *IL-1B (-511)* and the decreased risk of T2DM in the dominant model in the EA subgroup was convincing.

First, IL-1β was considered a mediator of fever, and rapidly, it was found that IL-1β induced innate immunity to defend against pathogens. However, chronic overexpression of IL-1β has been related to multiple immune diseases, including T2DM (Mandrup-Poulsen, 1996). Eventually, it was suggested that IL-1β can induce the inflammatory microenvironment of islets, leading to impaired insulin secretion, decreased cell proliferation and apoptosis of β-cells, ultimately contributing to the development of T2DM (Rhodes, 2005). Donath, M Y et al. extensively described the development of islet inflammation in GK rats, suggesting that the expression of pro-inflammatory cytokines IL-1β and others (IL-6, TNFα) was increased in islets, while elevated expression levels of many chemokines (CXCL1/KC, MCP-1, MIP-1α) and infiltration of immune cells in islets were observed (Ehses et al., 2009). In all cases, macrophage infiltration is increased in the islet inflammatory process (Ehses et al., 2007). Moreover, two independent studies have now indicated that the numbers of islet-related CD68^+^ cells are increased in T2DM patients (Ehses et al., 2007; Richardson et al., 2009). In addition, data obtained from laser-captured β-cells from T2DM patients have shown that evaluated expression levels of IL-1β and chemokines possibly led to this immune cell infiltration (Böni-Schnetzler et al., 2008). Since it can regulate various inflammatory processes, any change in the level of IL-1β in blood or tissue possibly affects these processes. Larsen et al. suggested that blocking IL-1β is a potential therapy in the treatment of T2DM (Larsen et al., 2007). The *IL-1B (-511) C* allele is associated with higher expression of IL-1β and with severe inflammation in the liver, while the *(-511) T* allele is associated with lower levels of IL-1β (Hirankarn et al., 2006; Vishnoi et al., 2008). In this meta-analysis, we found that the *IL-1B (-511) T* allele had a decreased T2DM risk in the EA subgroup, suggesting an influence of the *IL-1B (-511)* polymorphism system in this ethnic group.

The IL-1RN 2* allele (two repeats) is associated with increased IL-1Ra levels (Danis et al., 1995), which can compete with an inhibitor of IL-1β by binding to the IL-1 receptor. The 2* allele significantly increases the IL-1β secretion *in vitro* and balances the expression of IL-1β and IL-1Ra (Santtila, Savinainen & Hurme, 1998), and their ratio (IL-1Ra/IL-1β) determines the severity of inflammation. It has been shown that the IL-1RN 2* allele is associated with a low ratio (IL-1Ra/IL-1β), thereby inducing a longer and more severe pro-inflammation (Witkin, Gerber & Ledger, 2002). The frequency of the 2* allele is increased in inflammatory conditions or autoimmune diseases (Tountas et al., 1999; Van der Paardt et al., 2002). Consistently, this meta-analysis suggested that the IL-1RN 2* allele and 2*2* homozygotes increased T2DM risk, which indicated that the IL-1RN 2* allele and 2*2* homozygote polymorphism play critical roles in the development of T2DM.

Although we have devoted considerable efforts and resources to testing the potential association of the *IL-1B (-511)* and *IL-1RN* (VNTR) polymorphisms with T2DM risk, this analysis still has some limitations. First, significant heterogeneity was found in pooled analyses between the *IL-1B (-511)* polymorphism and T2DM risk. Due to the limited number of included publications, we conducted further ethnic stratification analysis and meta-regression to identify the exact sources of between-study heterogeneity. The meta-regression results showed that ethnicity was not the source of heterogeneity (both values of tau^2^ were not much different (0.34 *vs* 0.40)). Some possible relevant factors (genotyping method, sex) may lead to heterogeneity. However, subgroup analysis can significantly reduce heterogeneity. Second, the literature articles were primarily published in English or Chinese; thus, some eligible publications may be missing, causing some bias. Third, the publications included have some confounders, such as age, sex, diet, and exercise, whereas we used only the raw data to conduct pooled analysis; therefore, we were unable to control these possible confounders or test the potential gene-environment interactions. Fourth, the number of included publications was limited; thus, additional studies with large sample sizes and a wider variety of ethnicities are needed in the future to evaluate the association.

In conclusion, our meta-analysis first indicated that the *IL-1B (-511) T* allele polymorphism is associated with decreased T2DM risk in the EA population and that the *IL-1RN* 2* allele and 2*2 * homozygote polymorphism are strongly associated with increased T2DM risk. Further well-designed studies including different ethnicities with large sample sizes are needed to verify this conclusion.

##  Supplemental Information

10.7717/peerj.12384/supp-1 Supplemental Information 1 Supplemental Tables

10.7717/peerj.12384/supp-2 Supplemental Information 2 PRISMA checklist

10.7717/peerj.12384/supp-3 Supplemental Information 3 Systematic Review and Meta-Analysis Rationale

## Abbreviations

T2DM: type 2 diabetes mellitus
IL-1: Interleukin-1
IL-1Ra: IL-1 receptor antagonist
IL-1β: interleukin-1β
HWE: Hardy–Weinberg Equilibrium
OR: odds ratios
CIs: confidence intervals
EA: East Asian

## Additional Information and Declarations

### Data Availability

The following information was supplied regarding data availability: The raw data is available in Tables 1 and 2.
