# Supplementary material for: Association between IL-1B (-511)/IL-1RN (VNTR) polymorphisms and type 2 diabetes: a systematic review and meta-analysis
Source: PeerJ. 2021 Oct 25;9:e12384. doi: 10.7717/peerj.12384 (PMC8552784; doi:10.7717/peerj.12384)
Supplement: Supplemental Information 1 [file peerj-09-12384-s001.docx]

**Supplementary Materials:**

Table S1 The detailed scores for each part of NOS of each study

| Author | Year | QA Scores | | | |
| --- | --- | --- | --- | --- | --- |
|  |  | Selection | Comparability | Exposure | Total |
| Zhang Jian | 2004 | 4 | 1 | 2 | 7 |
| Zhang Ping-an | 2004 | 4 | 1 | 3 | 8 |
| Zhou Jian-zhong | 2010 | 3 | 1 | 3 | 7 |
| Petra Borilova Linhartova | 2019 | 4 | 1 | 3 | 8 |
| Alexandra I. F. Blakemore | 1995 | 3 | 1 | 3 | 7 |
| B.R Achyut | 2006 | 4 | 1 | 3 | 8 |
| Liu Chang | 2014 | 3 | 1 | 3 | 7 |
| Cao Yong | 2013 | 4 | 1 | 3 | 8 |
| Lin Neng-bo | 2016 | 4 | 1 | 3 | 8 |
| Natalie E. Doody | 2017 | 4 | 1 | 3 | 8 |
| Benja Muktabhant | 2013 | 4 | 1 | 2 | 7 |
| Safaa I. Tayel | 2018 | 4 | 1 | 3 | 8 |

QA: Quality Assessment

Table S2 The detailed scores for each part of STREGA in each study

| Author | Year | STREGA Scores | | | | | | |
| --- | --- | --- | --- | --- | --- | --- | --- | --- |
|  |  | Title and Abstract | Introduction | Methods | Results | Discussion | Other Information | Total |
| Zhang Jian | 2004 | 1 | 2 | 7 | 4 | 3 | 1 | 18 |
| Zhang Ping-an | 2004 | 1 | 2 | 7 | 4 | 2 | 1 | 17 |
| Zhou Jian-zhong | 2010 | 1 | 2 | 8 | 4 | 2 | 1 | 18 |
| Petra Borilova Linhartova | 2019 | 1 | 2 | 8 | 4 | 4 | 1 | 20 |
| Alexandra I. F. Blakemore | 1995 | 1 | 2 | 7 | 4 | 3 | 1 | 18 |
| B.R Achyut | 2006 | 1 | 2 | 8 | 5 | 4 | 1 | 21 |
| Liu Chang | 2014 | 1 | 2 | 6 | 3 | 4 | 1 | 17 |
| Cao Yong | 2013 | 1 | 2 | 7 | 4 | 3 | 1 | 18 |
| Lin Neng-bo | 2016 | 1 | 2 | 7 | 4 | 3 | 1 | 18 |
| Natalie E. Doody | 2017 | 1 | 2 | 6 | 4 | 3 | 1 | 17 |
| Benja Muktabhant | 2013 | 1 | 2 | 7 | 3 | 3 | 1 | 17 |
| Safaa I. Tayel | 2018 | 1 | 2 | 8 | 4 | 3 | 1 | 19 |

STREGA: STrengthening the REporting of Genetic Association studies
