# Supplementary material for: Association between IL-1B (-511)/IL-1RN (VNTR) polymorphisms and type 2 diabetes: a systematic review and meta-analysis
Source: PeerJ. 2021 Oct 25;9:e12384. doi: 10.7717/peerj.12384 (PMC8552784; doi:10.7717/peerj.12384)
Supplement: Supplemental Information 3 [file peerj-09-12384-s003.docx]

**Association between *IL-1B (-511)/IL-1RN (VNTR)* Polymorphisms and T2DM Risk: Evidence from a Meta-Analysis**

**Systematic Review and Meta-Analysis Rationale**

1. The rationale for conducting the meta-analysis

An increasing evidence have showed that metaflammation plays a vital role in the development of T2DM and its cardiovascular complications, and pro-inflammatory cytokines especially Interleukin-1β (IL-1β) plays a key role in this process. Meanwhile, the increased level of IL-1β in human pancreatic cells due to the evaluated glucose concentrations and decreased level of IL-1 receptor antagonist (IL-1Ra) in islets of T2DM patients result in impaired insulin secretion, decreased cell proliferation, and the apoptosis of β-cell. Therefore, the genetic polymorphisms which regulate the expression level of IL-1β and IL-1Ra might have an essential impact on the inter-individual differences in T2DM. While several case-control studies have illustrated the association between them, the conclusions remain controversial. In order to illustrate the potential association between the IL-1B (-511), IL-1RN (VNTR) polymorphism and the risk of T2DM, we conducted this meta-analysis of data reported in 12 publications.

2. The contribution that it makes to knowledge in light of previously published related reports, including other meta-analyses and systematic reviews

This is the first meta-analysis focusing on the association between the IL-1B (-511), IL-1RN (VNTR) polymorphism and the risk of T2DM. And we addressed the association of IL-1B (-511) and IL-1RN (VNTR) polymorphisms with T2DM risk. And our findings indicated that IL-1B (-511) C allele polymorphism is associated with decreasing T2DM risk in EA, and IL-1RN 2* allele, 2*2* homozygotes polymorphism are strongly associated with increasing T2DM risk. Considering the significant role of genetic factors in the development of T2DM, it is meaningful to identify the association of IL-1B (-511) and IL-1RN (VNTR) polymorphisms with T2DM risk.
